# Challenges of hemodialysis in Vietnam: experience from the first standardized district dialysis unit in Ho Chi Minh City

**DOI:** 10.1186/s12882-015-0117-2

**Published:** 2015-08-01

**Authors:** Cuong Minh Duong, Dariusz Piotr Olszyna, Phong Duy Nguyen, Mary-Louise McLaws

**Affiliations:** School of Public Health and Community Medicine, UNSW Medicine, UNSW Australia, Level 3 Samuels Building, Sydney, 2052 NSW Australia; Division of Infectious Diseases, University Medicine Cluster, National University Health System, Singapore, Singapore; Training Center for Family Physicians, University of Medicine and Pharmacy at Ho Chi Minh City, Ho Chi Minh City, Vietnam

**Keywords:** Hemodialysis, Challenges, Hepatitis B virus, Hepatitis C virus, Non-compliance

## Abstract

**Background:**

Hemodialysis is an increasingly common treatment in Vietnam as the diagnosis of end stage renal disease continues to rise. To provide appropriate hemodialysis treatment for end-stage renal disease patients, we conducted a 1-year cross-sectional study to measure the prevalence of bloodborne infection and factors associated with non-compliant behaviors in hemodialysis patients.

**Methods:**

One hundred forty-two patients were tested for hepatitis B virus (HBV) surface antigen and hepatitis C virus (HCV) core antigen. They provided demographic, medical and dialysis information. Non-compliant behaviors were obtained from their medical records.

**Results:**

Overall, 99 % of patients reused their dialyzers and 46 % had arteriovenous fistula on admission*.* Both HBV and HCV equally accounted for 8 % of patients and concurrent infection accounted for 1 %. Non-compliance rates of dietary and medication were 39 and 27 % respectively. 42 % of patients missed hemodialysis session, 8 % were verbally or physically abusive and 9 % were non-cooperative. Of the 54 % catheterized patients, 7 % improperly cared for their dialysis access. Dietary non-adherence was associated with male patients (*p* = 0.03) and medication non-adherence was associated with younger age (*p* = 0.05). Duration between diagnosis of chronic kidney disease and initiation of hemodialysis was associated with improper care of dialysis access (*p* = 0.04). Time on hemodialysis was associated with missed hemodialysis session (*p* = 0.007) and verbal or physical abuse (*p* = 0.01).

**Conclusion:**

Health services need to provide safe practice for dialyzer reuse given the endemicity of hepatitis. We believe a national survey similar to ours about seroprevalence and infection control challenges would prepare Vietnam for providing safer satellite treatment units. Safe hemodialysis services should also comprise patient preparedness, education and counseling.

## Background

End stage renal disease (ESRD) is managed in Vietnam by hemodialysis, peritoneal dialysis or kidney transplantation. Besides in-hospital care, several developed countries have introduced satellite hemodialysis centers and home-based hemodialysis [1, 2]. In Vietnam, in-hospital care provides hemodialysis, but now satellite units are also becoming one of the most common treatment modalities under the encouragement of the government [3]. Both approaches expand treatment access for ESRD patient and reduce the patient load in tertiary hospitals. Hemodialysis treatment for ESRD patient has commenced since 1983 in 2 largest cities, Ho Chi Minh City and Hanoi [4]. Patients covered the full cost of their treatment and only a small group being insured in accordance with their employment entitlements. Home-based hemodialysis is unlikely an optimal approach due to limited resources in providing hemodialysis facilities to approximately 80,000 patients needing daily treatment [5] as well as insufficient number of clean patients’ houses. Instead, the Ministry of Health has enhanced the number of satellite hemodialysis centers at district and provincial levels nationwide. Universal health insurance coverage of major hemodialysis costs ranged from 80 to 100 % [6]. Yet hospital-based and satellite hemodialysis units have still been facing several obstacles to provide a safe hemodialysis service due to limited number of trained personnel, no standardized infection prevention strategies associated with hemodialysis, and financial constraint to comply with treatment recommendations [7–9]. Other hardships are inadequate pre-dialysis care and consultation by nephrologist, as well as low adherence to treatment [10–14]. Our study was conducted at the first standardized hemodialysis district unit in Ho Chi Minh City to identify the challenges for safe hemodialysis treatment by measuring the patterns of patient’s non-compliance and their associated factors. Other outcomes included the prevalence of HBV and HCV infections.

## Methods

### Design of the study

Between November 2012 and November 2013, all patients aged 18 years and older, treated at the District-6 Hospital Hemodialysis Unit in Ho Chi Minh City, Vietnam were invited to participate in our cross-sectional study. There were 142 adult patients treated at the unit, and all patients provided written informed consent. The study protocol was approved by the Human Research Ethics Committee UNSW Australia (approval number HC12363), Ho Chi Minh City Health Service (approval number 3242/SYT-VP) and District 6 Hospital (approval number 223/TB-BV) authorities.

Participants provided demographic details on age, sex, occupation, home address and type of health insurance coverage. Medical and hemodialysis information were also obtained in a self-administered questionnaire and cross checked using medical records. Medical and hemodialysis information included symptoms on first admission, types of vascular access used for the first hemodialysis, frequency of intravenous iron therapy and blood transfusion, use of erythropoiesis-stimulating agents (ESAs), dialyzer reuse, the duration (in months) of hemodialysis and hemoglobin concentration (Hb). Blood bank records were unavailable and all patients who reported to have received a blood transfusion prior to admission were asked to provide the year, place and the number of transfusion.

Missed hemodialysis was defined as having missed at least one session in a month. Signing off a session early and late arrival to a session by at least 10 min in a month were defined as a shortened session. An extra hemodialysis treatment was determined as a patient requesting at least one session outside the treatment plan prescribed by the nephrologist. Verbal or physical abuse was defined as using inappropriate and disrespectful words or inflicting physical harm on staffs or patients in at least one occasion. Non-cooperative behavior was described as not following staffs’ instructions or breaking the clinic rules during hemodialysis sessions. For infection prevention and patient safety purposes, patients were required to follow their treatment schedule and the arteriovenous fistula (FAV) site must be cleaned with soap and water before arriving for treatment. Patients with temporary catheter were required to keep the catheter site and dressing clean and dry. Catheter site dressing must only be changed by healthcare workers. In case of emergency, patients were asked to contact the closest health clinic. Besides, no consumption of food was allowed and defecation was discouraged during hemodialysis sessions. Without prior permission, patients were not allowed to have relative, caregiver or children in the hemodialysis room. Verbal and physical abuse as well as uncooperative behavior were documented and obtained from medical records.

The study clinic provided medication for patients every fortnight consisting of ESAs and drugs to treat concurrent diseases and ESRD associated disorders. Patients were classified as non-compliant if they missed at least one dose of ESAs per fortnight. Patients were reported as non-adherent to medication if they missed medication at home or presented at the clinic one day later than scheduled at least once per fortnight. Receiving blood and intravenous iron transfusions were defined as having at least one blood transfusion and one dose of intravenous iron during the course of treatment. The study clinic provided a list of recommended food for all patients on admission. Patients were required to record a food diary for dietary assessment and also counselled individually to limit their fluid intake. Dietary non-adherence was described as consuming non-recommended foods at least once per week or gaining more than 2 kg between hemodialysis sessions. This information was obtained from the medical records.

Dialyzers were reprocessed with peroxyacetic acid-hydrogen peroxide mixture in the semi-automated system. The dialysis unit applied its own infection control policy and was in accordance with the national infection control precautions [15], the Renal Association [16] and CDC [17] recommendations. However, due to limited resources, there were modifications that were outside the recommendations [15–17] and have been detailed elsewhere [18].

### Laboratory tests

The presence of HCV core antigen and HBV surface antigen in blood samples were detected by the ARCHITECT HCV Ag assay and ARCHITECT HBsAg assay (ABBOTT Laboratories) which are chemiluminescent microparticle immunoassay.

### Statistical analysis

Data were managed and analyzed using Statistical Package for the Social Sciences (SPSS) version 20 (IBM). Continuous and categorical variables were presented as means ± one standard deviation (SD) and percentages. Chi-square and Fisher’s Exact test were used to calculate significance levels for categorical data. T-test and Mann–Whitney U test were utilized for the comparison of continuous data. The significance was set at *p* ≤0.05.

## Results

### Demographic, clinical and laboratory characteristics

In the 12-month period, the mean age of 142 enrolled patients was 55 years (SD 16, range 19–87 years) of whom 47 % were male and 33 % lived rurally (Table 1). The age distribution reflected younger patients with less than half (41 %, 58/142) of all patients being ≥60 years of age, 37 % (53/142) 41–59 years and 22 % (31/142) 19–40 years of age. Most patients, 55 % (78/142) were unemployed, 34 % (49/142) were employed, 8 % (12/142) engaged in home duties and 2 % (3/142) were students. Of the 49 patients employed 65 % (32/49) were employed as a casual worker, driver or farmer and 35 % (17/49) owned a small business. Chronic tiredness was the most common (72 %, 102/142) symptom on admission followed by edema (16 %, 23/142) and dyspnea (12 %, 17/142) (Table 2). The prevalence was 8 % (11/142) for HCV, 8 % (11/142) for HBV and 1 % (1/142) had a concurrent HCV and HBV infection. By the end of the study period the cumulative incidence for HCV (2/130) was 1.5 % and HBV (3/130) was 2.3 %. On admission, less than half (46 %, 65/142) of all patients had FAV as vascular access for hemodialysis, while 54 % (76/142) were already catheterized. The mean admission Hb was 8.8 g/dl (±1.5, range 3.7–13.1 g/dl). On admission or during the study period, 56 % (79/142) received at least one blood transfusion and 40 % (56/142) received an intravenous iron transfusion. Just over a third (37 %, 53/142) of patients commenced hemodialysis promptly after their chronic kidney disease (CKD) diagnosis, 22 % (32/142) after 1–10 months, 15 % after 11–20 (21/142) months and 25 % (36/142) after 20 months. Dialyzers were reused by nearly all patients (99 %, 140/142). Most patients (77 %, 109/142) had 80 % insurance coverage, 21 % (30/142) had 95 % coverage and only 2 % (3/142) were fully insured.

**Table 1:** Demographic characteristic of 142 patients

Male	47 % (67/142)
Living in rural area	33 % (47/142)
Age (years)	
Median (LQ; UQ)	55 (42; 67)
Mean (± SD)	55 (±16)
Range	19–87
Employment status	
Employed	34 % (49/142)
Unemployed	55 % (78/142)
In home duties	8 % (12/142)
Students	2 % (3/142)
Type of health insurance coverage	
100 %	2 % (3/142)
95 %	21 % (30/142)
80 %	77 % (109/142)

**Table 2:** Clinical and laboratory characteristics of 142 patients

Duration from diagnosis of chronic kidney disease to commencing hemodialysis (months)	
<1	37 % (53/142)
1–10	22 % (32/142)
11–20	15 % (21/142)
>20	25 % (36/142)
Symptoms at time of diagnosis of ESRD	
Chronic tiredness	72 % (102/142)
Edema	16 % (23/142)
Dyspnea	12 % (17/142)
Dialysis catheter access on admission	
Arteriovenous fistula (FAV)	46 % (65/142)
Femoral vein cannulation	36 % (52/142)
Non-tunneled internal jugular catheter	12 % (17/142)
Subclavian venous cannulation	3 % (4/142)
Tunneled internal jugular catheter	3 % (4/142)
Hemoglobin concentration on admission (g/dl)	
Median (LQ; UQ)	8.9 (7.9; 9.8)
Mean (± SD)	8.8 (±1.5)
Range	3.7–13.1
Treatment	
Having intravenous iron transfusion	40 % (56/142)
Having blood transfusion	56 % (79/142)
Patients reuse own dialyzer	99 % (140/142)
Duration of hemodialysis (months)	
Median (LQ; UQ)	27 (17; 47)
Mean (± SD)	40 (±40)
Range	1–245
Viral hepatitis infection status	
HCV infection	8 % (11/142)
HBV infection	8 % (11/142)
Concurrent HCV and HBV infections	1 % (1/142)

### Characteristics and determinants of non-compliant behaviors

There were 42 % (59/142) of patients who missed their hemodialysis session, 12 % (18/142) shortened their session and 11 % (16/142) required an extra treatment (Table 3). Over one third (39 %, 55/142) of patients reported non-adherence to dietary restrictions and 27 % (38/142) non-adherence to medication. During treatment sessions 9 % (12/142) of patients were non-cooperative while 8 % (11/142) were verbally or physically abusive. Of the 77 patients catheterized, 7 % (5/77) were classified as having improperly cared for their dialysis access.

**Table 3:** Non-compliant characteristics of 142 patients

Characteristics	% (N)
Missed at least one session	42 (59)
Require extra dialysis treatment	11 (16)
Early sign-off from session	6 (9)
Late arrival for session	6 (9)
Non-adherence to dietary restrictions	39 (55)
Non-adherence to medication	27 (38)
Non-cooperate with staffs	9 (12)
Verbal or physical abuse	8 (11)
Improper care of dialysis access (*n* = 77)	7 (5)

The mean duration of hemodialysis of patients who missed their sessions was lower than those who did not (29.1 SD 30 vs. 47 SD 45.1 months, *p* = 0.007). The mean duration of hemodialysis of patients who were verbally or physically abusive was higher than those who did not (60.2 SD 40.6 vs. 37.8 SD 40.1 months, *p* = 0.01). The mean age of patients who did not adhere to medication was lower than those who did (50.5 SD 16.5 vs. 56.4 SD 15.7 year-olds, *p* = 0.05). Dietary non-adherence was 2.07 time higher in men than women (95%CI OR 1.04–4.12). The mean duration between CKD diagnosis and hemodialysis initiation of patients who improperly cared for dialysis access was shorter than those who did not (0.2 SD 0.4 vs. 24.6 SD 39.9 months, *p* = 0.04).

There were no significant differences between patients who shortened their treatment session or required an extra treatment compared with those who did not with respect to: sex, age, duration of dialysis, and duration between CKD diagnosis and hemodialysis initiation.

## Discussion

### Demographic, clinical and laboratory characteristics

ESRD is not only a public health burden but also an economic burden [19]. Most of our patients were still of working age given the retirement age in Vietnam is 55 years old for female and 60 for male [20]. However, just less than three quarters of the surveyed participants were unemployed at the timed diagnosis of ESRD. The gross national income per capita is US$1730 [21], while the current cost for thrice-weekly hemodialysis treatment is on average $3600 per year ($25 per session). To encourage this treatment regimen, Vietnam national health insurance system covered 80 % of dialysis care in 1996 [6]. This proportion is now up to 100 % but only for certain patient groups such as revolutionary contributors and social protection group [22]. The government has also introduced a special insurance scheme for underprivileged households [6]. In our participants, 80 % coverage was the most common insurance type. Even in countries with good social health insurance system, such as Australia, a high rate of CKD patients has been found to have financial hardship [23]. Therefore, the gap in costs for hemodialysis, medical supply and medication remains important challenges for adherence by patients to their hemodialysis plan. To reduce costs, reprocessing dialyzers was a common practice. This has immeasurable consequences to patients’ health, economic security and Vietnam’s prosperity.

HCV and HBV infections pose a severe impact on patients’ health and financial issues. We found a prevalence of 8 % for each of HCV and HBV infection and 1 % for co-infection. The HCV and HBV prevalence among blood donors in the same region are just 0.8 and 3.7 % respectively [24]. This suggests that HCV and HBV infections are endemic in our hemodialysis patients. The associated complications not only impact on morbidity and mortality [25] but also incur treatment cost that patients cannot meet.

Given the government’s efforts to improve community health including the treatment and prevention of CKD, poor attitude towards health remains a major barrier. Self-medication accounts for three quarters of all episodes of illness each year in Vietnam [26]. Traditional care-seeking behavior is also popular among Vietnamese people [27]. Through our observations, patients usually utilize health services when having obvious illnesses or when self-mediation does not relieve their symptoms. Self-medication is more common among the elderly, low-income people or those who live in rural area [26]. As a further complication, this community also accesses to health services infrequently [26]. In addition, early detection of CKD requires a high level of awareness and knowledge of vague presentations in early stages of the disease among both the community and healthcare staffs [28, 29]. Several studies have shown a strong relationship between late nephrology referral, unplanned hemodialysis treatment and poor outcomes consisting of increased hospitalization rate, emergency hemodialysis, early death risk and more temporary vascular access [30, 31]. In contrast, survival in CKD patients is improved when treatment is provided by a nephrologist for at least 1 year before the initiation of hemodialysis [32]. We found more than half of our patients had been referred to a nephrologist and commenced hemodialysis within 10 months following their diagnosis of CKD. We did not collect the reasons for late presentation of CKD patients to the nephrologists yet this may be associated with common presentations of vague symptoms of CKD or the common practice of self-medication.

Late CKD diagnosis results in the ineffective treatment to delay progression to end stage. This also induced insufficient preparation for the life-long hemodialysis including timely placement of FAV. The most common presenting symptoms in our study were chronic tiredness, edema and dyspnea. These are usual symptoms of ESRD complications comprising anemia, hypervolemia and pulmonary edema [33, 34]. Indeed, more than half of our patients had a Hb concentration of less than the recommended level, 9 g/dl [35], and more than one-third needed emergency hemodialysis on admission. Therefore, ESAs, blood and intravenous iron transfusion along with emergency hemodialysis were immediately commenced to stabilize the patients’ severe condition. Preparation for the first hemodialysis vascular access was still problematic since over half of patients had been using temporary vascular access instead of FAV. This resulted in an increase in hospitalization and length of hospital stay during the first hemodialysis treatment. Although hemodialysis services are being decentralized in Vietnam, several district and provincial centers are still unable to perform FAV operation due to limited surgical resources. As a result, patients are referred to tertiary hospitals where they are charged a high treatment fee.

### Characteristics and determinants of non-compliant behaviors

It is accepted that treatment non-compliance adversely affects patient outcomes and augments healthcare costs [36]. Apart from self-harm, noncompliant behavior impacts the normal work-load of the hemodialysis unit [37]. Noncompliance with treatment plan in our patients was problematic as nearly half of patients missed their sessions while 11 % required extra treatment and 12 % shortened their sessions. Young age, male and longer duration on hemodialysis have been reported to be associated with skipping and shortening the dialysis sessions [12, 36]. We found that duration of hemodialysis of patients missing dialysis sessions was lower than that for patients who did not miss any sessions while age and gender were not related to missing sessions. Patients new to dialysis were not always compliant with their treatment plan. The reason for non-compliance was not examined but personal observations suggested that this may be due to lack of mental and physical preparation and the discomfort derived from the first hemodialysis sessions. These issues can be addressed during pre-education program in the early diagnosis stage. After a long treatment duration, patients’ perception towards the efficacy of hemodialysis improved. In view of this, a pre-dialysis preparation program is essential to enhance treatment compliance. In 2012, Chan YM et al. emphasized the effect of financial constraint on patients’ compliance [12]. We did not assess the relationship between patients’ financial status and treatment adherence but we noted that most of our patients were unemployed. This financial hardship may have attributed to their noncompliance with treatment plan.

Non-adherence to dietary restrictions and medication were present in 39 % and 27 % of our patients. We found a relationship between older age and medication adherence. In addition, dietary non-adherence was more common among male than female patients. These associations were also reported elsewhere [12, 38].

Since patients were not well-prepared for hemodialysis including timely placement of permanent vascular access [39], catheterization was common during their first treatments. This made professional instruction about care of the temporary vascular access crucial. In this study patients with shorter duration between CKD diagnosis and hemodialysis initiation were more likely to inappropriately care for their dialysis access. It is not surprising patients referred to nephrologists earlier had better preparedness for hemodialysis treatment.

Verbal and physical abuse and non-cooperation during treatment were not conducive to a safe working environment or treatment conditions [37]. Staffs were ill-prepared for abuse and as might respond improperly in such situations [40]. Our patients who were verbally or physically abusive had longer duration of hemodialysis than patients who were not. Junior nurses have more frequent patient contact than senior ones and as a result report having experienced abuse from patients more often [41]. In our study we found that patients with a longer history of hemodialysis had more contact with healthcare workers over a long period of time than new patients. In some unfavorable conditions, such as being made to wait for treatments patients often responded aggressively to instructions. Our nurses reported more verbal and physical abuse than physicians. One explanation may be patients were less likely to be aggressive with physicians if they believed treatment could be withheld [41].

## Conclusions

Chronic hemodialysis will continue to be a burden for patients and health service providers in Vietnam. Given the endemicity of hepatitis health services need to provide safe practice for dialyzer reuse. We believe a national survey similar to ours about seroprevalence and infection control challenges would prepare Vietnam for providing safer satellite treatment units. Safe hemodialysis services should also comprise patient preparedness, education and counseling.

### Study limitations and strengths

The study was conducted at a typical satellite hemodialysis unit in Vietnam and as such the results are limited to these types of health services. Reasons for patients’ non-compliance to the recommended treatment regime and reasons for aggressive behavior were not collected. But this information could provide insight to strategies for improving the service.

Our clinic utilizes both paper-based and electronic medical records which allow recording collection of patients’ health related activities in real-time. The study data were obtained from handwritten medical records and then cross-checked with electronic ones. Any discrepancies were reviewed and verified to ensure the validity of data.
